# Editorial: Research in Transgender Healthcare: What Have We Learned and Where Are We Going?

**DOI:** 10.3389/fendo.2021.832866

**Published:** 2022-01-07

**Authors:** Rosa Fernández, Sarah M. Burke

**Affiliations:** ^1^ Laboratory of Psychobiology, Department of Psychology, Institute Advanced Scientific Research Center (CICA), University of A Coruña, A Coruña, Spain; ^2^ University Medical Center Groningen, Department of Psychiatry, Interdisciplinary Center for Psychopathology and Emotion Regulation, University of Groningen, Groningen, Netherlands

**Keywords:** cisgender, gender dysphoria, gender incongruence, gender-affirming hormonal treatment, healthcare, non-binary, transgender

Gender incongruence (GI) is defined as “an individual’s discontent with their assigned gender and their identification with a gender other than that associated with their birth sex based on physical sex characteristics” (1).

The origin of GI appears to be complex and multifactorial. From the extensive research that has been conducted over the past few years, three main factors have been identified as key mechanisms for understanding GI: genes, hormones, and the environment.

Accordingly, our Frontiers Research Topic includes twelve articles that cover very varied topics about GI, including cardiovascular effects of treatment, surgical outcomes, new treatment options and healthcare quality in a broader sense. This research involved the hard work of sixty-six authors that was carried out mainly in Europe (Austria, Belgium, Germany, Italy, Serbia, and Spain), Australia, and also the United States.

An interesting study about the prevalence of cardiometabolic risk factors in transgender individuals receiving feminizing therapy for ≥6 months was carried out by Balcerek et al. in a sample of 296 transgender individuals. The authors found that a greater proportion of trans individuals ≥45 years of age were treated with transdermal estradiol. Of those who received oral estradiol, the median dose was lower. Importantly, relatively higher doses of estradiol put people at risk of cardiometabolic diseases, especially in combination with older age. The authors point out that the most prevalent cardiometabolic risk factor in the ≥45 years group was hypertension (29%), followed by current smoking (24%), obesity (20%), dyslipidaemia (16%) and diabetes (9%).

On a related topic, Totaro et al., in a meta-analytic study, analyzed the risk of venous thromboembolism (VTE) in transgender people undergoing feminizing hormone treatment. The overall pooled prevalence estimate for VTE was 2%, but with a large heterogeneity across studies. A number of factors could contribute to the variable VTE risk in transgender people undergoing gender-affirming hormone treatment (GAHT), including the type of estrogen and the route of administration, age at the estrogen therapy onset, treatment duration, concomitant conditions such as smoking, obesity, thrombophilia and other comorbidities.

This topic of cardiovascular health outcomes due to GAHT was also examined by Aranda et al. The authors present a review of the current literature on the effects of GAHT, in both transgender men and transgender women, on various cardiovascular health outcomes.

An opinion paper included in our Research Topic addresses a clever approach on increasing inclusiveness in studies on Traumatic Brain Injury (TBI). The authors, Duncan and Garijo-Garde emphasize the importance of studying the influence of sex, gender and sex hormones in the TBI population. They highlight the need for evidence-based guidelines, and clinically translatable diagnostic and prognostic models.

The current endocrinological treatment guidelines serve primarily those who wish to transition from male to female or vice versa. But non-binary individuals currently make up more than 25% of the transgender population (2). Thus, there is a dearth of hormonal options for those who identify as non-binary and seek an androgynous appearance that is neither overtly masculine nor feminine. Regarding this topic, a potential option for non-binary GAHT was presented by Xu et al. In this article, the authors discuss the theoretical use of selective estrogen receptor modulators (SERMs) for non-binary persons assigned male at birth seeking an androgynous appearance through partial feminization without breast growth. The authors concluded that an individualized decision is needed when considering the use of SERMs in non-binary people, with recognition of their experimental nature, significant potential risks, and the urgent need for more research.

In our Research Topic, we also include a case report by Pang et al. who present the use of minoxidil as an alternative treatment option to testosterone administration for transgender men who desire an increase in facial hair growth.

Bordas et al. report on a follow-up study of surgical outcomes in 813 transgender men. The authors evaluated outcomes of metoidioplasty after on average 94 months post-operation. Urethroplasty was found to be without complications in 81% to 90% of the cases, and 99% of the patients who answered the questionnaire were mainly to fully satisfied with the surgical result.

GAHT may be recommended in many cases and it has been shown to improve many facets of transgender individuals’ functioning. In a prospective longitudinal controlled study, Foster Skewis et al. examined the effects of GAHT on quality of life of a group of transgender individuals over a 6-month period. The authors concluded that in transgender people initiating masculinizing or feminizing treatments, there was a decrease in gender dysphoria experienced and a clinically significant improvement in emotional well-being and social functioning aspects of quality of life, relative to a cisgender comparison group.

A systematic review of the barriers to accessing transgender healthcare in rural regions was carried out by Renner et al. The authors found an overrepresentation of transgender people in vulnerable socioeconomic situations, primarily due to experiences of discrimination. At the same time, rural or suburban living areas often lack specialized trans-related healthcare. Together, the lack of both socioeconomic resources and access to transgender healthcare can exacerbate health-related distress and impairment for transgender individuals.

A systematic review and a global expert survey in 39 countries about centralized (i.e., one interdisciplinary team) and decentralized (different medical institutions) delivery of transgender healthcare services was carried out by Koehler et al. The authors shed light on this important aspect of transgender healthcare and gained valuable knowledge for the further improvement of transgender healthcare quality.

Finally, in the work of Guethlein et al., the authors compare issues related to health and healthcare of transgender people in Germany with those in other European countries. The authors review the care offered by specialized centers with regard to treatment of and support for transgender people.

Approaching the topic of GI from a much different angle, Ramirez et al. address the question of the origins of GI with an epigenetics study. Both genetic, as well as (social and physical) environmental factors, have been found to play a role in the development of human gender, including transgender identity by directly or indirectly altering gene expression and behavior. In this respect, epigenetics offers many research opportunities, especially with regard to GI, as they reflect the interconnection between genes, hormones, and the environment.

The main finding of that study was that, prior to GAHT, the transgender group had a different global CpG methylome compared to the cisgender sample. This suggests that there are differences at the methylation level between cis- and transgender individuals even before GAHT. Furthermore, these findings indicate that not only genetics, but also epigenetics could be implicated in the biological basis of GI.

When the authors compared individuals assigned male at birth (cis vs. trans), they found significant differences in the methylation level of 22 CpGs. However, with respect to individuals assigned female at birth, significant differences in methylation of only 2 CpGs were found. Thus, there were larger differences in methylation level between the male-assigned groups (cis vs. trans) than between the female-assigned groups (cis vs trans). Furthermore, one of these CpGs, related to the *MPPED2* gene, was shared by both transgender men and women, suggesting a similar underlying mechanism for both sexes. Among the CpGs differing between the cis- and transgender samples, at least four genes were clearly involved in brain development and neurogenesis. These findings support the view that combining genetic and epigenetic approaches in parallel may be a successful way to better understand the mechanisms underlying sex- and gender identity related brain development.

## Author Contributions

All authors listed have made a substantial, direct, and intellectual contribution to the work and approved it for publication.

## Funding

This study was supported by the Spanish Ministry of Science and Innovation Grant No. PGC2018-094919-B-C22.

## Conflict of Interest

The authors declare that the research was conducted in the absence of any commercial or financial relationships that could be construed as a potential conflict of interest.

## Publisher’s Note

All claims expressed in this article are solely those of the authors and do not necessarily represent those of their affiliated organizations, or those of the publisher, the editors and the reviewers. Any product that may be evaluated in this article, or claim that may be made by its manufacturer, is not guaranteed or endorsed by the publisher.

## References

[1] Butler G. Gender Incongruence. Paediatr Child Health (2020) 30(12):407–10. doi: 10.1016/j.paed.2020.09.001

[2] Cheung AS Leemaqz SY Wong JWP Chew D Ooi O Cundill P. Non-Binary and Binary Gender Identity in Australian Trans and Gender Diverse Individuals. Arch Sex Behav (2020) 49(7):2673–81. doi: 10.1007/s10508-020-01689-9 32285311

